# The Hydrophobicity and Antifungal Potentiation of Burkholdine Analogues

**DOI:** 10.3390/molecules27041191

**Published:** 2022-02-10

**Authors:** Hiroyuki Konno, Mio Sasaki, Hinata Sano, Keima Osawa, Kazuto Nosaka, Shigekazu Yano

**Affiliations:** 1 Department of Biological Engineering, Graduate School of Science and Engineering, Yamagata University, Yonezawa 992-8510, Japan; sasaki.mio25@gmail.com (M.S.); tyh52030@st.yamagata-u.ac.jp (H.S.); txw83003@st.yamagata-u.ac.jp (K.O.); shige-y@yz.yamagata-u.ac.jp (S.Y.); 2 Faculty of Pharmaceutical Science, Mukogawa Women’s University, Nishinomiya 663-8179, Japan; knosaka@mukogawa-u.ac.jp

**Keywords:** antifungal activity, cyclic octalipopeptide, burkholdine, potentiation effect

## Abstract

The burkholdines are a family of cyclic lipopeptides reported to exhibit antifungal activity. We synthesized a series of 18 burkholdine analogues in good yield by conventional Fmoc-SPPS followed by cyclization with DIPCI/HOBt in the solution phase. Although none of the synthesized peptides exhibited antifungal activity, several did potentiate the antibiotic effect of the antibiotic G418, including the Thr-bearing Bk analogue (**4b**) and the tartaramide-bearing Bk analogue (**5b**). This work exemplifies the potential of burkholdine analogues as potentiating agents.

## 1. Introduction

The burkholdines (Bks) are a family of 25-membered cyclic lipopeptides composed of eight amino acids arranged in a lactam ring bearing a lipophilic side chain, which were first isolated from the proteobacteria *Burkholderia ambifaria 2.2N* by Schmidt in 2010 [1,2,3,4]. All naturally occurring Bks isolated so far have demonstrated potent antifungal activity, especially Bk-1119, whose activity is up to 25 times higher than that of amphotericin B [5,6,7]. The mechanism of this activity is thought to entail the inhibition of fungal β-glucan synthase, as is the case for the candine antibiotics micafungin [8] (MIC_90_ = 0.015 µg/mL (*Candida albicans*)) and caspofungin [9] (MIC_90_ = 0.25 µg/mL (*C. albicans*)) [10,11]. Bk-1119 is a promising lead compound for the development of safer antifungal drugs because it targets a β-glucan synthase not found in humans. However, neither the active site nor target enzyme of the Bks has been definitively identified, nor has the total synthesis of any Bk ever been reported. The role of the unusual residues β-OHTyr and β-OHAsn present in the Bk lactam ring is also unknown, but they are highly consequential, as their instability in strongly acidic solutions precludes the synthesis of peptides that incorporate them by conventional SPPS, which relies on TFA [12,13,14]. Accordingly, the scalable synthesis of Bks is needed to clarify their mechanism of action, establish the structural moieties essential for their good activity, and to generate enough material for mechanistic studies.

In our laboratory, we have been conducting structure–activity relationship studies of the Bks using Bk-1097 (**1**) as a lead compound [15,16]. Our studies so far have yielded two main findings. The first is that the 25-membered octapeptide structure is important for antifungal activity, although 27-membered octapeptides are also weakly active. The second is that the unusual amino acids present in the Bks are not essential for antifungal activity; analogue (**2**), which incorporates L-Tyr, L-Asn, and *N*-lauryl-3-amino-4-carbamoylpropanoic acid (LAP) in place of the unusual amino acids β-hydroxy Tyr, β-hydroxy Asn, and 3-amino-5,6,7-trihydroxyoctadecanoic acid (ATHOD) present in Bk-1097 (**2**), respectively, showed MIC = 25 µg/mL (*S. cerevisiae*). Herein, we report the synthesis and evaluation of Ser/Thr-type Bk analogues (**3**, **4**, **6**, and **7**) and hydrophobic, tartaramide-type Bk analogues (**5** and **8**).

## 2. Results and Discussions

Our previous work has established the importance of 25- and 27-membered rings for antifungal activity, showing that Bk analogues incorporating β,γ-diaminobutanoic acid (Dab) residues (the Xaa8 position) of the S configuration had better activity than their epimers—even though the natural Bk products are composed exclusively of amino acids of the R configuration [16].

**Figure 1:**
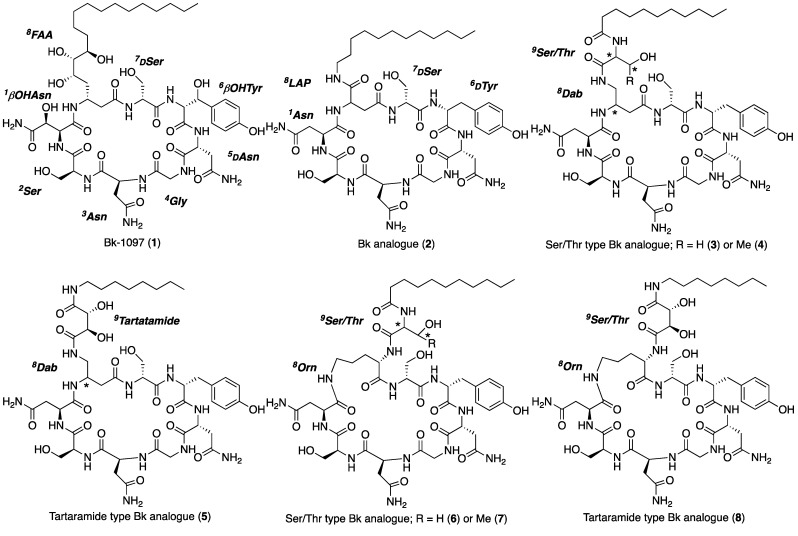
Bk-1097 (**1**) and Bk-1097 analogues (**2**–**8**).

**Scheme 1:**
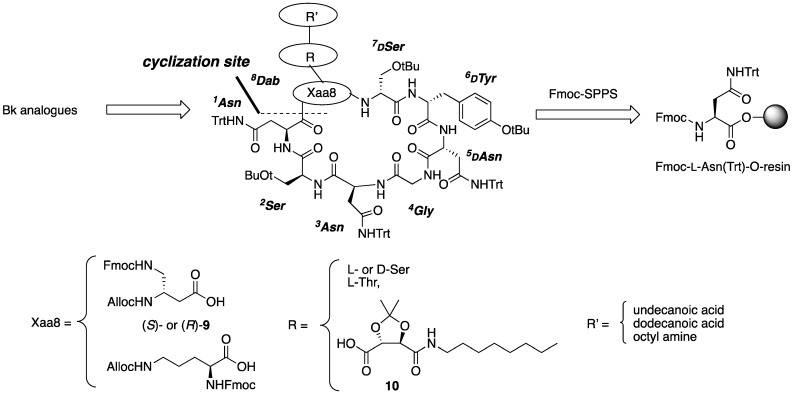
Synthesis of burkholdine analogues.

For this work, we aimed to synthesize two series of 25- and 27-membered analogues incorporating either enantiomer of Dab at the Xaa8 position, as well as a third series incorporating L-Orn to investigate the efficiency of the ring size. To assess the influence of the length of the fatty side chain on antifungal activity, Bk analogues bearing undecane-, dodecane-, and octane-based side chains were all designed within each series. Thus, we designed the target peptides (**3a**–**8**) (Figure 1, Scheme 1, Table 1).

First, we prepared (*S*) and (*R*)-g-Fmoc-b-Alloc-diaminobutanoic acids (Dab) (**9**), Fmoc-Orn(Alloc)-OH, and tartaramide derivative (Tat) (**10**). (*S*)- and (*R*)- g-Fmoc- b-Alloc-diaminobutanoic acids (Dab) (**9**) were synthesized as previously described by our group [16]. Fmoc-Orn(Alloc)-OH was derived from Fmoc-Orn(Boc)-OH in two steps. Tat derivative (**10**) was prepared in five steps from L-(+)-tartaric acid (see Supplementary Materials Figure S1).

Next, the designed Bk analogues were synthesized according to our previous synthetic strategy [12,13]. Macrolactamization between the Xaa8 amine and the C-terminus carboxylic acid of the linear peptides was accomplished over 14–62 h in the presence of DIPC/HOBt. Global deprotection was performed with TFA/TIPS/H_2_O (95:2.5:2.5). The corresponding linear peptides were prepared by Fmoc-SPPS on 2-chlorotrityl resin (2-CT resin) starting from the loading of Fmoc-Asn(Trt)-OH onto the C-terminus of the corresponding linear peptides. The Alloc groups protecting the branched amino acids on Xaa8 were removed using catalytic Pd(0) and the corresponding linear peptides bearing protected side chains achieved cleavage from resin with 20% HFIP/CH_2_Cl_2_ (Scheme 1).

**Scheme 2:**
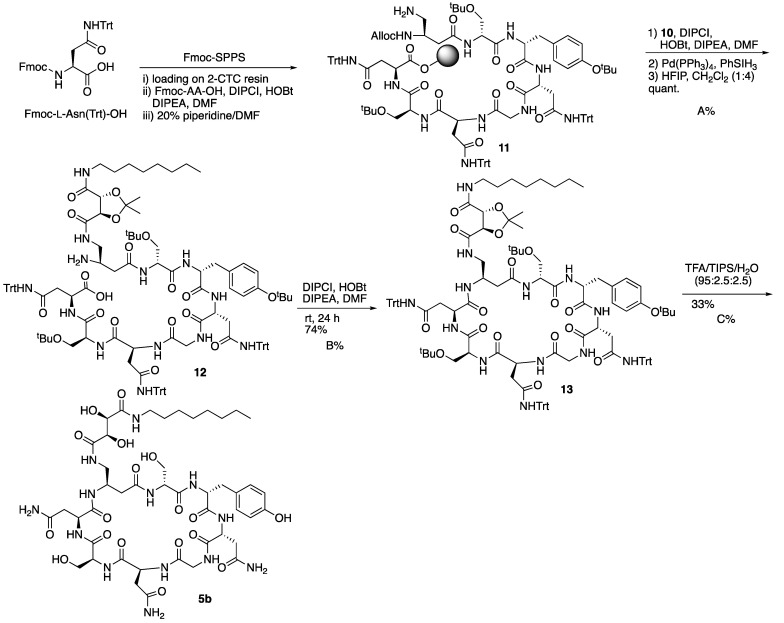
Synthesis of Bk analogue (**5b**).

The synthesis of **5b** is depicted in Scheme 2 and is representative of the procedure used for all the analogues. Fmoc-SPPS was performed to give the requisite linear peptide resin **11**, which was coupled with Tat derivative (**10**) in the presence of DIPCI/HOBt/DIPEA. Pd(0)-mediated Alloc deprotection was carried out to give the linear peptide **12** in excellent yield after cleavage from resin using 20% HFIP/CH_2_Cl_2_. Cyclization of the linear peptide **12** with DIPCI/HOBt for 24 h proceeded to give the cyclic peptide **13** in 74% yield. Finally, global deprotection of the cyclic peptide **13** by TFA/TIPS/H_2_O (95:2.5:2.5) gave Bk analogue **5b** in 33% yield after the purification by RP-HPLC. The overall yield of **5b** from resin loading was 25%, confirming the efficiency of the cyclization and the stability of **5b** under acidic conditions (Scheme 2). Reactions were monitored by RP-HPLC and measured by ESI-MS (Table 2 and Supplementary Materials Figure S1).

Analogues (**3a**)–(**8**) were prepared based on the synthesis of **5b**; the chemical yields of linear, cyclic, and designed peptides (A, B, and C%) and overall yields (D%) are shown in Table 2. Yields of the requisite linear peptides incorporating the Dab residue by Fmoc-SPPS were satisfactory (A%); those of the Orn-containing peptides (**6a**–**d**, **7**, and **8**) were moderated. Macrolactamization of the linear peptides was accomplished according to our optimized conditions (B%). Purification of crude protected cyclic peptides was performed by silica gel column chromatography. The efficiency of the cyclization step was noted to be influenced by both the sequence of the amino acids of the linear precursor and their stereochemistry. The final deprotection of the cyclic peptides also gave various yields. However, the cyclic peptides incorporating a (*R*)-Dab residue (**3e**–**5b**) were obtained in moderate yields, confirming the stability of peptides (**3e**–**5b**) under acidic conditions. Yields for steps A–D for each peptide are presented in Table 2. HPLC profiles and ESI-MS spectra data are included in the Supplementary Materials Figure S1. (Table 3).

The hydrophilicity of all of the cyclic peptides synthesized (**3a**)–(**8**) was investigated using RP-HPLC under the same eluting conditions (30–60% MeCN/H_2_O for 30 min); the corresponding retention times are shown in Table 3. In all cases, the retention times of the (*S*)-Dab-containing peptides were shorter than those of the (*R*)-Dab-containing peptides, which were similar to those bearing L-Orn. The retention times of the cyclic peptides were shorter for Tat > D-Ser > L-Thr > L-Ser, while peptides bearing the undecanoic acid moiety eluted faster than those bearing the decanoic acid by about 2 min.

In addition, the polar surface areas (PSA) and logP values of these peptides were calculated using SPARTAN’18 (Wavefunction), using their stable conformers as calculated using molecular mechanics. The PSA of the Tat-bearing cyclic peptides (**5a**) was found to be similar to that of Bk-1097 (**1**). The logP values of these peptides also showed a similar tendency to the PSA values. The calculated PSA and logP values of synthesized peptides (**3a**)–(**8**) were all negative and unlikely to exhibit cell penetration. Accordingly, any biological activities they exhibited almost certainly arose from their interactions with the cell surface.

The antifungal activities of Bk analogues (**3a**)–(**8**) were also evaluated at a concentration of 200 mg/mL. Against the yeast *S. cerevisiae* (ATCC204504), only **3b** and **3f** showed inhibitory effects, with MIC values of 100 and 200 mg/mL, respectively—far higher than that of Bk analogue (**2**) (MIC = 25 µg/mL) [16]. None of the peptides inhibited *A. oryzae* (NBRC100959) or *Candida viswanathii* (NBRC10321) at 200 mg/mL concentration. This lack of activity may have been due to the high hydrophobicities of the analogues precluding their entry into the cells; the PSA and logP values for the analogues were all higher than those of Bk analogues (**2**). Future work will include a structure activity–relationship study of natural Bk products and their analogues to better understand the mechanism of the antifungal inhibition (Table 3).

Two antibiotics with different mechanisms of action can have a synergistic effect, together exerting an antifungal activity higher than the sum of their individual activities. Accordingly, the potentiation [17,18] of the antifungal effect of G418 (Geneticin) [19,20], an aminoglycosyl-type antibiotic that inhibits protein biosynthesis by binding to the ribosome 70S and 80S subunits, and zeocin (phleomucin D1) [21,22], a glycopeptide antibiotic and DNA intercalator, by cyclic peptides (**3a**)–(**8**) was evaluated. Although the relatively high PSA and negative logP values of Bk analogues (**3a**)–(**8**) are not conductive to cell penetration, there was the possibility that their fatty side chains have an affinity for cell surfaces. A dose of each 100 mg/mL of the cyclic peptides (**3a**)–(**8**) did indeed potentiate the effect of G418 against the pathogenic fungus *S. cerevisiae*; the use of **3g**, **4b**, **5b**, **6c**, **7**, and **8** dropped the MIC value of G418 from 25 mg/mL to 12.5 mg/mL, a two-fold increase in potency. The mechanism of action is not clear, but it is possible that the interaction of the cyclic peptides (**3a**)–(**8**) with the cell wall increases its susceptibility to penetration by G418. However, no potentiation of zeocin by the cyclic peptides (**3a**)–(**8**) was observed, perhaps because it proved a highly potent inhibitor on its own (MIC = 3.13 mg/mL) (Table 4).

## 3. Materials and Methods

### 3.1. General

All solvents were reagent grade (Nacalai tesque, Kyoto, Japan and Kishida Chemical, Osaka, Japan). All commercial reagents were of the highest purity available (Watanabe Chemical, Hiroshima, Japan, Fujifilm-Wako, Tokyo, Japan and TCI, Tokyo, Japan). Optical rotations were determined with a JASCO P-2200 polarimeter at the sodium D line (Tokyo, Japan). IR spectra were recorded on a Spectrum Two instrument (PerkinElmer, Waltham, MA, USA). ^1^H (500 and 600 MHz) values were determined on a Jeol JNM-ECX500 and JNM-ECX-600 (Tokyo, Japan). Chemical shifts are reported in ppm with reference to Me_4_Si [^1^H-NMR: TMS (0.00)] or solvent signals [^1^H-NMR: CDCl_3_ (7.26), ^13^C-NMR: CDCl_3_ (7.26)]. Mass spectra were obtained using Jeol AccuTOF JMS-T100LC (ESI-MS) (Tokyo, Japan). Analytical TLC was performed on Merck silica gel 60F_254_ (Darmstadt, Germany). Crude products were purified by column chromatography on silica gel 60 N (Kanto, particle size, (spherical, neutral) at 63–210 μm (Tokyo, Japan). Analytical HPLC was carried out using a system comprising a Shimadzu (Kyoto, Japan) SPD-10A UV-Vis detector, Hitachi (Tokyo, Japan) L-6000 Pump and Hitachi L-6200 Intelligent Pump all equipped with a Cosmosil 5C_18_-AR-II column (4.6 ID × 150 mm, Nacalai tesque, Kyoto, Japan) eluting with a linear gradient of 0.1% TFA/MeCN (*B* solution) in 0.1% TFA/H_2_O (*A* solution) over at a run time of 30 min (flow rate of 1 mL/min). Preparative HPLC was performed using a Shimadzu SPD-10A*i* UV–Vis detector and Hitachi L-6200 intelligent pump all equipped with a Cosmosil 5C_18_-AR-II column (10ID × 250 mm) eluting with a linear gradient of 0.1% TFA/MeCN (*B* solution) in 0.1% TFA/H_2_O (*A* solution) over a run time of 30 min (flow rate of 2 mL/min). UV measurements were recorded at a wavelength of 220 nm for the peptide analyses.

### 3.2. Fmoc-Orn(Alloc)-OH

4N HCl/AcOEt (20 mL, A00024, Watanabe Chemical, Japan) was added to Fmoc-Orn(Boc)-OH (300 mg, 0.660 mmol, K00450, Watanabe Chemical, Japan) and the mixture was stirred for 1.5 h. The reaction mixture was concentrated in vacuo. The residue was dissolved in dioxane/H_2_O (1:1 *v*/*v*) (6.6 mL), K_2_CO_3_ (365 mg, 2.64 mmol, 28508-94, Nacalai tesque, Japan) was added, and the mixture was cooled to 0 °C. AllocCl (210 µL, 1.99 mmol, 2937-50-0, Sigma-Aldrich, Tokyo, Japan) was added dropwise to the mixture. The reaction mixture was warmed to room temperature and stirred. After 1 h, H_2_O was added, the pH was adjusted to 7 with saturated NH_4_Cl aq., and the mixture was diluted with AcOEt and extracted. The organic layer was washed with brine, dried with anhydrous MgSO_4_, filtered, then the solvent was evaporated under reduced pressure. The residue was purified by silica gel chromatography (CHCl_3_-MeOH = 98:2 *v*/*v*) to give Fmoc-Orn(Alloc)-OH (200 mg, 0.456 mmol, 69%) as a white solid. ^1^H-NMR (500 MHz, CDCl_3_): δ 1.43–1.73 (4H, m), 3.15 (2H, m), 4.20 (1H, t, *J* = 6.8 Hz), 4.40 (2H, d, *J* = 6.5 Hz), 4.54 (2H, d, *J* = 4.0 Hz), 5.20 (1H, d, *J* = 10 Hz), 5.28 (1H, d, *J* = 17 Hz), 5.89 (1H, m), 7.29 (2H, t, *J* = 7.0 Hz), 7.38 (2H, t, *J* = 6.5 Hz), 7.59 (2H, d, *J* = 7.0 Hz), 7.75 (2H, d, *J* = 7.5 Hz).

### 3.3. (R,R)-Diethyl 2,3-O-isopropylidentartrate

L-(+)-Tartaric acid (1.00 g, 6.66 mmol, 203-00052, Fujifilm-Wako, Japan) was dissolved in MeOH (22.2 mL) and cooled to −80 °C. SOCl_2_ (1.5 mL, 20.7 mmol, 206-01103, Fujifilm-Wako, Japan) was added dropwise to the solution. The mixture was stirred for 3 h and was allowed to warm to room temperature. After 12.5 h, the reaction solution was concentrated in vacuo to obtain a colorless oil, which was dissolved in CH_2_Cl_2_ (22.2 mL). 2.2-Dimethoxypropane (5.4 mL, 43.8 mmol, 042-06963, Fujifilm-Wako, Japan) and *p*-TsOH·H_2_O (634 mg, 3.33 mmol, 34208-05, Nacalai tesque, Japan) were added and the mixture was stirred with refluxing (45 °C). After 4 h, the mixture was warmed to room temperature and saturated acrylamide (10 mL, 00807-05, Nacalai tesque, Japan) was added slowly. AcOEt and H_2_O were added, the mixture was extracted with AcOEt, the organic layer was washed with brine, then it was dried over anhydrous MgSO_4_ and filtered and the solvent was evaporated under reduced pressure. The residue was purified by silica gel chromatography (hexane-AcOEt = 8:1 *v*/*v*) to give (*R*,*R*)-diethyl 2,3-*O*-isopropylidentartrate (1.11 g, 5.07 mmol, 76%, 2 steps) as a yellow oil. ^1^H-NMR (600 MHz, CDCl_3_) δ 4.73 (s, 2H), 3.75 (s, 6H), 1.41 (s, 6H). The spectral data are in perfect agreement with the report by Hilpert et al.

### 3.4. (4R,5R)-2,2-Dimethyl-5-[(octylamino)carbonyl]-1,3-dioxolane-4-carboxylic Acid (***10***)

(*R*,*R*)-Diethyl 2,3-*O*-isopropylidentartrate (207 mg, 0.949 mmol) was dissolved in MeCN-H_2_O (7:93 *v*/*v*, 23.6 mL) at 0 °C. Then, 0.2 M KOH (5.7 mL, 1.14 mmol, 28616-45, Nacalai tesque, Japan) was added to the mixture over 1 h. After 30 min, H_2_O and Et_2_O were added to the solution, extracted, and the pH of the aqueous layer was adjusted to pH 3 with 1 M HCl aq. The aqueous layer was extracted with Et_2_O. The organic layer was washed with brine, dried over anhydrous MgSO_4_, filtered, and the solvent was removed under reduced pressure to give a pale yellow oil (154 mg, 0.754 mmol, 80%). The oily substance (501 mg, 2.45 mmol) was dissolved in DMF (8.2 mL). Octyl amine (916 µL, 5.53 mmol, 150-00173, Fujifilm-Wako, Japan), Et_3_N (1.02 mL, 7.36 mmol, 34804-85, Nacalai tesque, Japan), BOP (3.26 g, 7.37 mmol, A00045, Watanabe Chemical, Japan), and HOBt· H_2_O (1.13 g, 7.38 mmol, A00014, Watanabe Chemical, Japan) were added to the solution. After 2.5 h, the reaction mixture was diluted with H_2_O and AcOEt, extracted, washed with brine, dried over anhydrous MgSO_4_, filtered, and the solvent was evaporated under reduced pressure. The residue was purified by silica gel chromatography (hexane-AcOEt = 4:1 *v*/*v*) to give the octyl amide (678 mg, 2.15 mmol, 88%) as a clear oil. To a solution of the octyl amide in *t*BuOH-H_2_O (1:1 *v*/*v*, 7.2 mL) we added LiOH· H_2_O (181 mg, 4.30 mmol, 20635-22, Naacalai tesque, Japan). After 2 h, the mixture was added to H_2_O and Et_2_O, extracted, and the pH of the aqueous layer was adjusted to pH 3 with 1 M HCl. The aqueous layer was extracted with Et_2_O and combined organic layers were washed with brine, dried over anhydrous MgSO_4_, and filtered, then the solvent was concentrated under reduced pressure to give **10** (633 mg, 2.10 mmol, 98%) as an oil. ^1^H NMR (600 MHz, CDCl_3_): δ 6.81 (br, 1H), 4.52 (q, *J* = 9.4 Hz, 2H), 3.30–3.45 (m, 2H), 1.48–1.60 (m, 8H), 1.24–1.32 (m, 10H), 0.87 (t, *J* = 6.9 Hz, 3H). ^13^C-NMR (150 MHz, CDCl_3_): δ 171.4, 167.9, 113.1, 39.8, 31.8, 29.2, 26.8, 26.1, 22.7, 14.2. IR (KBr) νmax cm^−1^: 3313, 2916, 2850, 1736, 1604, 1561. ESI-MS: calcd. for C_15_H_27_NO_5_ [M + H]^+^ 302.20, 302.11 [α]_D_^21^ = 24.1° (*c* = 1.02, CHCl_3_); mp: 80–81 °C.

### 3.5. Cyclo-[Undecanoic Acid-Ser-(S)-7-D-Ser-D-Tyr-D-Asn-Gly-Asn-Ser-Asn] (***5b***)

2-Chlorotrityl chloride resin (102 mg, 81.6 µmol, A00330, Watanabe Chemical, Japan) was swelled by shaking in DMF (2 mL) for 30 min; Fmoc-Asn(Trt)-OH (147 mg, 246 µmol, K00900, Watanabe Chemical, Japan), DIPEA (28.0 µL, 164 µmol, 053-05355, Fujifilm-Wako, Japan), and DMF (1.5 mL) were added and the mixture was shaken for 2 h; 20% Piperidine (A00176, Watanabe Chemical, Japan)/DMF was added and shaken for 30 min; Fmoc-Ser(*t*Bu)-OH (94.2 mg, 246 µmol, K00458, Watanabe Chemical, Japan), DIPCI (38.0 µL, 245 µmol, A00011, Watanabe Chemical, Japan), HOBt· H_2_O (37.5 mg, 245 µmol), DIPEA (28.0 µL, 164 µmol), and DMF(1.5 mL) were added and shaken for 2 h; 20% Piperidine/DMF was added and shaken for 30 min; Fmoc-Asn(Trt)-OH (147 mg, 246 µmol), DIPCI (38.0 µL, 245 µmol), HOBt· H_2_O (37.8 mg, 247 µmol), DIPEA (28.0 µL, 164 µmol), and DMF(1.5 mL) were added and shaken for 2 h; 20% Piperidine/DMF was added and shaken for 30 min; Fmoc-Gly-OH (73.3 mg, 247 µmol, K00434, Watanabe Chemical, Japan), DIPCI (38.0 µL, 245 µmol), HOBt· H_2_O (37.7 mg, 246 µmol), DIPEA (28.0 µL, 164 µmol), and DMF(1.5 mL) were added and it was shaken for 2 h; 20% Piperidine/DMF was added and shaken for 30 min; Fmoc-D-Asn(Trt)-OH (147 mg, 246 µmol, K00909, Watanabe Chemical, Japan, DIPCI (38.0 µL, 245 µmol), HOBt· H_2_O (38.0 mg, 248 µmol), DIPEA (28.0 µL, 164 µmol), and DMF(1.5 mL) were added and shaken for 3 h; 20% Piperidine/DMF was added and shaken for 30 min; Fmoc-D-Tyr(*t*Bu)-OH (113 mg, 246 µmol, K00468, Watanabe Chemical, Japan), DIPCI (38.0 µL, 245 µmol), HOBt· H_2_O (37.5 mg, 245 µmol), DIPEA (28.0 µL, 164 µmol), and DMF(1.5 mL) were added and shaken for 3 h; 20% Piperidine/DMF was added and shaken for 30 min; Fmoc-D-Ser(*t*Bu)-OH (94.2 mg, 246 µmol, K00459, Watanabe Chemical, Japan), DIPCI (38.0 µL, 245 µmol), HOBt· H_2_O (38.0 mg, 248 µmol), DIPEA (28.0 µL, 164 µmol), and DMF(1.5 mL) were added and shaken for 3 h; 20% Piperidine/DMF was added and shaken for 30 min; (*S*)-**9** (104 mg, 245 µmol), DIPCI (38.0 µL, 245 µmol), HOBt· H_2_O (37.8 mg, 247 µmol), DIPEA (28.0 µL, 164 µmol), and DMF(1.5 mL) were added and shaken for 2.5 h; 20% Piperidine/DMF was added and shaken for 30 min; Fmoc-Ser(*t*Bu)-OH (94.0 mg, 245 µmol, K00458, Watanabe Chemical, Japan), DIPCI (38.0 µL, 245 µmol), HOBt· H_2_O (37.6 mg, 246 µmol), DIPEA (28.0 µL, 164 µmol), and DMF(1.5 mL) were added and shaken for 2 h; 20% piperidine/DMF was added and shaken for 30 min; Undecanoic acid (45.9 mg, 246 µmol, 218-00042, Fujifilm-Wako, Japan), DIPCI (38.0 µL, 245 µmol), HOBt· H_2_O (38.1 mg, 249 µmol), DIPEA (28.0 µL, 164 µmol), and DMF(1.5 mL) were added and it was shaken for 5 h; Pd(PPh_3_)_4_ (94.8 mg, 82.0 µmol, T1350, TCI, Japan), PhSiH_3_ (98.6 µL, 816 µmol, P1291, TCI, Japan) and DMF/CH_2_Cl_2_ (1:1 *v*/*v*, 1.6 mL) were added, and the mixture was shaken for 3 h. After washing the resin with Et_2_O, 20% HFIP/dry CH_2_Cl_2_ (6.0 mL) was added and the mixture was shaken for 2 h. The resin was filtered while being washed with dry CH_2_Cl_2_ and the filtrate was concentrated. When Et_2_O was added to the mixture, crystals were precipitated. After centrifugation (4 °C, 7000 rpm, 5 min), the process of removing the supernatant was performed three times. The obtained crystals were air-dried to obtain a crude protected linear peptide (137 mg, 66.5 µmol, overall 81%) as reddish-brown crystals. DIPCI (64.4 µL, 416 µmol), HOBt· H_2_O (63.9 mg, 417 µmol), DIPEA (42.8 µL, 250 µmol), and DMF (11.1 mL) were added to the linear peptide and the mixture was shaken for 24 h. H_2_O was added and the mixture was extracted with AcOEt. A pale yellow protected cyclic peptide (58.9 mg, 28.8 µmol, 43%) was obtained by silica gel chromatography (CHCl_3_-MeOH = 90:10 *v*/*v*). TFA (A00026, Watanabe Chemical, Japan)/TIPS (T1533, TCI, Japan)/H_2_O (95:2.5:2.5 *v*/*v* 3.0 mL) was added to cyclic peptide and the mixture was stirred for 2 h. When the reaction solution was concentrated while being azeotropically boiled with dry CH_2_Cl_2_ and Et_2_O was added, white crystals were precipitated. After centrifugation (4 °C, 7000 rpm, 5 min), the process of removing the supernatant was performed three times. The obtained crystals were air-dried and purified by RP-HPLC (Cosmosil 5C18-AR-II (10 × 250 mm), with 30 to 70% *B* in *A* over 30 min, 2.0 mL/min, *t*_R_ = 17.4 min) with freeze-drying, after which a white crystalline cyclic peptide (**3a**) (2.30 mg, 2.11 µmol) was obtained in a total yield of 3%. Linear peptide: HPLC: Cosmosil 5C_18_-AR-Ⅱ (4.6 × 150 mm), 70 to 100% *B* in *A* over 30 min, 1.0 mL/min, *t*_R_ = 22.6 min. ESI-MS: calcd. for C_120_H_149_N_13_O_18_ [M + H]^+^ 2061.12, 2060.79; cyclic peptide: HPLC: Cosmosil 5C_18_-AR-Ⅱ (4.6 × 150 mm), 70 to 100% *B* in *A* over 30 min, 1.0 mL/min, *t*_R_ = 38.4 min. ESI-MS: calcd. for C_120_H_147_N_13_O_17_ [M + H]^+^ 2043.11, 2042.86; **5b**: HPLC: Cosmosil 5C_18_-AR-Ⅱ (4.6 × 150 mm), 30 to 70% *B* in *A* over 30 min, 1.0 mL/min, *t*_R_ = 9.3 min. ESI-MS: calcd. for C_47_H_73_N_13_O_17_ [M + H]^+^ 1092.53, 1092.23.

#### 3.5.1. Cyclo-[Dodecanoic Acid-Ser-(S)-7-D-Ser-D-Tyr-D-Asn-Gly-Asn-Ser-Asn] (**3b**)

A (195 mg) = quant. HPLC: 70 to 100% *B* in *A* over 30 min, 1.0 mL/min, *t*_R_ = 23.8 min. ESI-MS: calcd. for C_121_H_151_N_13_O_18_ [M + H]^+^ 2075.14, found 2074.88.

B (52.6 mg, 25.6 µmol) = 32%, HPLC: 70 to 100% *B* in *A* over 30 min, 1.0 mL/min, *t*_R_ = 42.8 min. ESI-MS: calcd. for C_121_H_149_N_13_O_17_ [M + H]^+^ 2057.13, found 2056.78.

C (3.30 mg, 2.98 µmol) = 12%, D = 4%. HPLC: 30 to 70% *B* in *A* over 30 min, 1.0 mL/min, *t*_R_ = 11.7 min. ESI-MS: calcd. for C_48_H_75_N_13_O_17_ [M + H]^+^ 1106.55, found 1106.29.

#### 3.5.2. Cyclo-[Undecanoic Acid-D-Ser-(S)-7-D-Ser-D-Tyr-D-Asn-Gly-Asn-Ser-Asn] (**3c**)

A (234 mg) = quant. HPLC: 70 to 100% *B* in *A* over 30 min, 1.0 mL/min, *t*_R_ = 22.6 min. ESI-MS: calcd. for C_120_H_149_N_13_O_18_ [M + H]^+^ 2061.12, found 2060.84.

B (74.6 mg, 36.5 µmol) = 45%. HPLC: 70 to 100% *B* in *A* over 30 min, 1.0 mL/min, *t*_R_ = 38.1 min. ESI-MS: calcd. for C_120_H_147_N_13_O_17_ [M + H]^+^ 2043.11, found 2042.80.

C (3.20 mg, 2.93 µmol) = 8% D = 4% HPLC: 30 to 70% *B* in *A* over 30 min, 1.0 mL/min, *t*_R_ = 9.4 min. ESI-MS: calcd. for C_47_H_73_N_13_O_17_ [M + H]^+^ 1092.53, found 1092.27.

#### 3.5.3. Cyclo-[Dodecanoic Acid-D-Ser-(S)-7-D-Ser-D-Tyr-D-Asn-Gly-Asn-Ser-Asn] (**3d**)

A = (195 mg) = quant. HPLC: 70 to 100% B in A over 30 min, 1.0 mL/min, *t*_R_ = 24.6 min. ESI-MS: calcd. for C_121_H_151_N_13_O_18_ [M + H]^+^ 2075.14, found 2074.88.

B (44.3 mg, 21.5 µmol) = 27%, HPLC: 70 to 100% *B* in *A* over 30 min, 1.0 mL/min, *t*_R_ = 41.9 min. ESI-MS: calcd. for C_121_H_149_N_13_O_17_ [M + H]^+^ 2057.13, found 2056.89.

C (5.50 mg, 4.97 µmol) = 23%, D = 6%, HPLC: 30 to 70% *B* in *A* over 30 min, 1.0 mL/min, *t*_R_ = 11.8 min. ESI-MS: calcd. for C_48_H_75_N_13_O_17_ [M + H]^+^ 1106.55, found 1106.29.

#### 3.5.4. Cyclo-[Undecanoic Acid-Thr-(S)-7-D-Ser-D-Tyr-D-Asn-Gly-Asn-Ser-Asn] (**4a**)

A (123 mg, 59.0 µmol) = 74%. HPLC: 70 to 100% B in A over 30 min, 1.0 mL/min, *t*_R_ = 23.9 min. ESI-MS: calcd. for C_121_H_151_N_13_O_18_ [M + H]^+^ 2075.14, found 2074.83.

B (54.9 mg, 26.7 µmol) = 45%. HPLC: 70 to 100% B in A over 30 min, 1.0 mL/min, *t*_R_ = 39.0 min. ESI-MS: calcd. for C_121_H_149_N_13_O_17_ [M + H]^+^ 2057.13, found 2056.83

C (1.80 mg, 1.63 µmol) = 6%, D = 2%. HPLC: 30 to 70% B in A over 30 min, 1.0 mL/min, *t*_R_ = 10.4 min. ESI-MS: calcd. for C_48_H_75_N_13_O_17_ [M + H]^+^ 1106.55, found 1106.29.

#### 3.5.5. Cyclo-[(2R,3R)-2,3-dihydroxy-4-(octylamino)-4-oxobutanoic Acid-(S)-7-D-Ser-D-Tyr-D-Asn-Gly-Asn-Ser-Asn] (**5a**)

A (185 mg = quant. HPLC: 70 to 100% *B* in *A* over 30 min, 1.0 mL/min, *t*_R_ = 17.5 min. ESI-MS: calcd. for C_117_H_141_N_13_O_19_ [M + H]^+^ 2033.05, found 2032.80.

B (41.9 mg, 20.8 µmol) = 26%. HPLC: 70 to 100% *B* in *A* over 30 min, 1.0 mL/min, *t*_R_ = 29.5 min. ESI-MS: calcd. for C_117_H_139_N_13_O_18_ [M + H]^+^ 2015.04, found 2014.83.

C (6.60 mg, 6.11 µmol) = 29%, D = 8%. HPLC: 30 to 70% *B* in *A* over 30 min, 1.0 mL/min, *t*_R_ = 5.3 min. ESI-MS: calcd. for C_45_H_69_N_13_O_18_ [M + H]^+^ 1080.50, found 1080.23.

#### 3.5.6. Cyclo-[Undecanoic Acid-Ser-(R)-7-D-Ser-D-Tyr-D-Asn-Gly-Asn-Ser-Asn] (**3e**)

A (242 mg) = quant. HPLC: 70 to 100% *B* in *A* over 30 min, 1.0 mL/min, *t*_R_ = 22.7 min. ESI-MS: calcd. for C_120_H_149_N_13_O_18_ [M + H]^+^ 2061.12, found 2060.79.

B (54.9 mg, 26.9 µmol) = 34%. HPLC: 70 to 100% *B* in *A* over 30 min, 1.0 mL/min, *t*_R_ = 42.3 min. ESI-MS: calcd. for C_120_H_147_N_13_O_17_ [M + H]^+^ 2043.11, found 2042.82.

C (8.50 mg, 7.78 µmol) = 29%, D = 10%. HPLC: 30 to 70% B in A over 30 min, 1.0 mL/min, *t*_R_ = 9.4 min. ESI-MS: calcd. for C_47_H_73_N_13_O_17_ [M + H]^+^ 1092.53, found 1092.27.

#### 3.5.7. Cyclo-[Dodecanoic Acid-Ser-(R)-7-D-Ser-D-Tyr-D-Asn-Gly-Asn-Ser-Asn] (**3f**)

A (234 mg) = quant. HPLC: 70 to 100% *B* in *A* over 30 min, 1.0 mL/min, *t*_R_ = 24.2 min. ESI-MS: calcd. for C_121_H_151_N_13_O_18_ [M + H]^+^ 2075.14, found 2074.83.

B (69.6 mg, 33.8 µmol) = 42%. HPLC: 70 to 100% *B* in *A* over 30 min, 1.0 mL/min, *t*_R_ = 43.7 min. ESI-MS: calcd. for C_121_H_149_N_13_O_17_ [M + H]^+^ 2057.13, found 2057.85.

C (11.5 mg, 10.4 µmol) = 31%, D = 13%. HPLC: 30 to 70% *B* in *A* over 30 min, 1.0 mL/min, *t*_R_ = 11.8 min. ESI-MS: calcd. for C_48_H_75_N_13_O_17_ [M + H]^+^ 1106.55, found 1106.29.

#### 3.5.8. Cyclo-[Undecanoic Acid-D-Ser-(R)-7-D-Ser-D-Tyr-D-Asn-Gly-Asn-Ser-Asn] (**3g**)

A (230 mg) = quant. HPLC: 70 to 100% *B* in *A* over 30 min, 1.0 mL/min, *t*_R_ = 23.8 min. ESI-MS: calcd. for C_120_H_149_N_13_O_18_ [M + H]^+^ 2061.12, found 2060.84.

B (40.7 mg, 19.9 µmol) = 25%. HPLC: 70 to 100% *B* in *A* over 30 min, 1.0 mL/min, *t*_R_ = 42.7 min. ESI-MS: calcd. for C_120_H_147_N_13_O_17_ [M + H]^+^ 2043.11, found 2043.71.

C (5.50 mg, 4.61 µmol) = 23%, D = 6%. HPLC: 30 to 70% *B* in *A* over 30 min, 1.0 mL/min, *t*_R_ = 9.6 min. ESI-MS: calcd. for C_47_H_73_N_13_O_17_ [M + H]^+^ 1092.53, found 1092.27.

#### 3.5.9. Cyclo-[Dodecanoic Acid-D-Ser-(R)-7-D-Ser-D-Tyr-D-Asn-Gly-Asn-Ser-Asn] (**3h**)

A (160 mg, 77.0 µmol) = 96%. HPLC: 70 to 100% *B* in *A* over 30 min, 1.0 mL/min, *t*_R_ = 25.8 min. ESI-MS: calcd. for C_121_H_151_N_13_O_18_ [M + H]^+^ 2075.14, found 2074.77.

B (71.8 mg, 34.9 µmol) = 45%. HPLC: 70 to 100% *B* in *A* over 30 min, 1.0 mL/min, *t*_R_ = 51.3 min. ESI-MS: calcd. for C_121_H_149_N_13_O_17_ [M + H]^+^ 2057.13, found 2057.80.

C (14.7 mg, 12.8 µmol) = 37%, D = 16%. HPLC: 30 to 70% *B* in *A* over 30 min, 1.0 mL/min, *t*_R_ = 11.8 min. ESI-MS: calcd. for C_48_H_75_N_13_O_17_ [M + H]^+^ 1106.55, found 1106.35.

#### 3.5.10. Cyclo-[Undecanoic Acid-Thr-(R)-7-D-Ser-D-Tyr-D-Asn-Gly-Asn-Ser-Asn] (**4b**)

A (181 mg) = quant. HPLC: 70 to 100% *B* in *A* over 30 min, 1.0 mL/min, *t*_R_ = 23.7 min. ESI-MS: calcd. for C_121_H_151_N_13_O_18_ [M + H]^+^ 2075.14, found 2074.77.

B (111 mg, 53.8 µmol) = 67%. HPLC: 70 to 100% *B* in *A* over 30 min, 1.0 mL/min, *t*_R_ = 41.2 min. ESI-MS: calcd. for C_121_H_149_N_13_O_17_ [M + H]^+^ 2057.13, found 2056.78.

C (20.4 mg, 18.4 µmol) = 34%, D = 23%. HPLC: 30 to 70% *B* in *A* over 30 min, 1.0 mL/min, *t*_R_ = 10.7 min. ESI-MS: calcd. for C_48_H_75_N_13_O_17_ [M + H]^+^ 1106.55, found 1106.35.

#### 3.5.11. Cyclo-[(2R,3R)-2,3-dihydroxy-4-(octylamino)-4-oxobutanoic Acid-(R)-7-D-Ser-D-Tyr-D-Asn-Gly-Asn-Ser-Asn] (**5b**)

A (182 mg) = quant. HPLC: 70 to 100% *B* in *A* over 30 min, 1.0 mL/min, *t*_R_ = 17.8 min. ESI-MS: calcd. for C_117_H_141_N_13_O_19_ [M + H]^+^ 2033.05, found 2032.70.

B (121 mg, 59.8 µmol) = 74%. HPLC: 70 to 100% *B* in *A* over 30 min, 1.0 mL/min, *t*_R_ = 31.2 min. ESI-MS: calcd. for C_117_H_139_N_13_O_18_ [M + H]^+^ 2015.04, found 2015.68.

C (21.6 mg, 20.0 µmol) 33%, D = 25%. HPLC: 30 to 70% *B* in *A* over 30 min, 1.0 mL/min, *t*_R_ = 6.4 min. ESI-MS: calcd. for C_45_H_69_N_13_O_18_ [M + H]^+^ 1080.50, found 1080.28.

#### 3.5.12. Cyclo-[Undecanoic Acid-Ser-Orn-D-Ser-D-Tyr-D-Asn-Gly-Asn-Ser-Asn] (**6a**)

A (128 mg, 61.8 µmol) = 56%. HPLC: 70 to 100% *B* in *A* over 30 min, 1.0 mL/min, *t*_R_ = 22.4 min. ESI-MS: calcd. for C_121_H_151_N_13_O_18_ [M + H]^+^ 2075.13, found 2074.95.

B (19.8 mg, 9.62 µmol) = 16%. HPLC: 70 to 100% *B* in *A* over 30 min, 1.0 mL/min, *t*_R_ = 38.1 min. ESI-MS: calcd. for C_121_H_149_N_13_O_17_ [M + H]^+^ 2057.12, found 2056.98.

C (2.09 mg, 1.89 µmol) = 20%, D = 2%. HPLC: 30 to 70% *B* in *A* over 30 min, 1.0 mL/min, *t*_R_ = 9.7 min. ESI-MS: calcd. for C_48_H_75_N_13_O_17_ [M + H]^+^ 1106.18, found 1106.43.

#### 3.5.13. Cyclo-[Dodecanoic Acid-Ser-Orn-D-Ser-D-Tyr-D-Asn-Gly-Asn-Ser-Asn] (**6b**)

A (70.6 mg, 33.8 µmol) = 63%. HPLC: 70 to 100% *B* in *A* over 30 min, 1.0 mL/min, *t*_R_ = 23.2 min. ESI-MS: calcd. for C_122_H_153_N_13_O_18_ [M + H]^+^ 2089.15, found 2089.15.

B (23.3 mg, 11.2 µmol) = 33%. HPLC: 70 to 100% *B* in *A* over 30 min, 1.0 mL/min, *t*_R_ = 40.9 min. ESI-MS: calcd. for C_122_H_151_N_13_O_17_ [M + H]^+^ 2071.14, found 2070.80.

C (1.17 mg, 1.04 µmol) = 9%, D = 2%. HPLC: 30 to 70% *B* in *A* over 30 min, 1.0 mL/min, *t*_R_ = 12.1 min. ESI-MS: calcd. for C_49_H_77_N_13_O_17_ [M + H]^+^ 1120.56, found 1120.34.

#### 3.5.14. Cyclo-[Undecanoic Acid-D-Ser-Orn-D-Ser-D-Tyr-D-Asn-Gly-Asn-Ser-Asn] (**6c**)

A (221 mg) = quant. HPLC: 70 to 100% *B* in *A* over 30 min, 1.0 mL/min, *t*_R_ = 22.8 min. ESI-MS: calcd. for C_121_H_151_N_13_O_18_ [M + H]^+^ 2075.13, found 2076.08.

B (128 mg, 62.0 µmol) = 77%. HPLC: 70 to 100% *B* in *A* over 30 min, 1.0 mL/min, *t*_R_ = 36.4 min. ESI-MS: calcd. for C_121_H_149_N_13_O_17_ [M + H]^+^ 2057.12, found 2057.09.

C (34.7 mg, 31.4 µmol) = 5%, D = 39%. ESI-MS: calcd. for C_48_H_75_N_13_O_17_ [M + H]^+^ 1116.18, found 1106.39.

#### 3.5.15. Cyclo-[Dodecanoic Acid-D-Ser-Orn-D-Ser-D-Tyr-D-Asn-Gly-Asn-Ser-Asn] (**6d**)

A (144 mg, 68.8 µmol) = 86%. HPLC: 70 to 100% *B* in *A* over 30 min, 1.0 mL/min, *t*_R_ = 23.6 min. ESI-MS: calcd. for C_122_H_153_N_13_O_18_ [M + H]^+^ 2089.15, found 2089.20.

B (77.8 mg, 37.5 µmol) = 55%. HPLC: 70 to 100% *B* in *A* over 30 min, 1.0 mL/min, *t*_R_ = 40.0 min. ESI-MS: calcd. for C_122_H_151_N_13_O_17_ [M + H]^+^ 2071.14, found 2070.77.

C (4.57 mg, 4.99 µmol) = 11%, D = 5%. HPLC: 30 to 70% *B* in *A* over 30 min, 1.0 mL/min, *t*_R_ = 11.2 min. ESI-MS: calcd. for C_49_H_77_N_13_O_17_ [M + H]^+^ 1120.56, found 1120.32.

#### 3.5.16. Cyclo-[Undecanoic Acid-Thr-Orn-D-Ser-D-Tyr-D-Asn-Gly-Asn-Ser-Asn] (**7a**)

A (45.7 mg, 21.8 µmol) = 41%. HPLC: 70 to 100% *B* in *A* over 30 min, 1.0 mL/min, *t*_R_ = 22.9 min. ESI-MS: calcd. for C_122_H_153_N_13_O_18_ [M + H]^+^ 2089.15, found 2089.12.

B (37.2 mg, 17.9 µmol) = 82%. HPLC: 70 to 100% *B* in *A* over 30 min, 1.0 mL/min, *t*_R_ = 38.7 min. ESI-MS: calcd. for C_122_H_151_N_13_O_17_ [M + H]^+^ 2071.14, found 2070.53.

C (2.09 mg, 1.87 µmol) = 10%, D = 3%. HPLC: 30 to 70% *B* in *A* over 30 min, 1.0 mL/min, *t*_R_ = 10.6 min. ESI-MS: calcd. for C_49_H_77_N_13_O_17_ [M + H]^+^ 1120.56, found 1120.25.

#### 3.5.17. Cyclo-[(2R,3R)-2,3-Dihydroxy-4-(octylamino)-4-oxobutanoic Acid-Orn-D-Ser-D-Tyr-D-Asn-Gly-Asn-Ser-Asn] (**8**)

A (105 mg, 51.3 µmol) = 39%. HPLC: 70 to 100% *B* in *A* over 30 min, 1.0 mL/min, *t*_R_ = 17.1 min. ESI-MS: calcd. for C_118_H_143_N_13_O_19_ [M + H]^+^ 2047.06, found 2047.24.

B (51.9 mg, 25.6 µmol) = 49%. HPLC: 70 to 100% *B* in *A* over 30 min, 1.0 mL/min, *t*_R_ = 30.9 min. ESI-MS: calcd. for C_118_H_141_N_13_O_18_ [M + H]^+^ 2029.05, found 2028.72.

C (4.44 mg, 4.06 µmol) = 16%, D = 3%. HPLC: 30 to 70% *B* in *A* over 30 min, 1.0 mL/min, *t*_R_ = 5.8 min. ESI-MS: calcd. for C_46_H_71_N_13_O_18_ [M + H]^+^ 1094.50, found 1094.29.

### 3.6. MIC Assay

#### 3.6.1. Preparation of YPD Medium

Bacto yeast extract (0.63 g, 70161, Fluka, Barcelona, Spain), peptone (1.00 g, 26421, Mikuni, Japan), and glucose (1.00 g, 16806-25, Nacalai tesque, Japan) were added to H_2_O (50 mL) at room temperature and the mixture was heated to 60 °C. After 30 min, the mixture was sterilized by heating in an autoclave at 121 °C for 20 min.

#### 3.6.2. Preparation of Test Fungus Culture Solution

DMSO (8 µL, 13406-55, Nacalai tesque, Japan) was added to YPD medium (750 µL). The test fungus culture solution (3 µL) was added to this solution (1.5 mL).

#### 3.6.3. Preparation of Inhibitor

The inhibitor (each 1.0 mg; **3a**–**h**, **4a**–**b**, **5a**–**b**, **6a**–**b**, **7**, **8**) was dissolved in DMSO (40 µL).

#### 3.6.4. Assay of Antifungal Activity

The prepared test bacteria (50 µL) were placed in A1–A12 of a 96-well plate. Bk analogue (50 µL) was added to A1, pipetting was performed, and then 50 µL was extracted from A1 and placed in A2. This operation was repeated until A11, and 50 µL extracted from A11 was discarded. The well plate was closed and incubated at 30 °C. After 24 h, the MIC values were evaluated.

#### 3.6.5. Potentiation Assay

The prepared test bacteria (50 µL) were placed in well A1–A12 of a 96-well plate. G418 (200 mg/mL, 108321-42-2, Nacalai tesque, Japan) in H_2_O, then medium (50 mL) was added to A1, pipetting was performed, and 50 µL was extracted from A1 and placed in A2. This operation was repeated until A11, and 50 µL extracted from A11 was discarded. After 30 min, Bk analogue (200 μg/mL) in medium (50 mL) was added to each solution (final concentration = 100 μg/mL). The well plate was closed and incubated at 30 °C. After 24 h, the MIC and potentiation values were evaluated.

## 4. Conclusions

We synthesized 18 cyclic Bk analogues via ordinary Fmoc-SPPS, macrolactamization, and global deprotection in solution phase. The antifungal activities of these cyclic peptides were very weak in comparison with the Bk analogue (**2**), but the Thr-bearing Bk analogue (**4b**) and Tat-bearing Bk analogue (**5b**) had weak potentiating effects on G418 and their syntheses were accomplished in good yield. We will investigate the optimized structures for potent potentiation activity and mechanisms of action in due course.

## Figures and Tables

**Table 1:** Molecular design of Bk-1097 analogues.

Compounds	Xaa8	R	R’
**3a**	(*S*)-Dab	L-Ser	Undecanoic acid
**3b**			Dodecanoic acid
**3c**		D-Ser	Undecanoic acid
**3d**			Dodecanoic acid
**4a**		L-Thr	Undecanoic acid
**5a**		L-(+)-Tat	Octyl amine
**3e**	(*R*)-Dab	L-Ser	Undecanoic acid
**3f**			Dodecanoic acid
**3g**		D-Ser	Undecanoic acid
**3h**			Dodecanoic acid
**4b**		L-Thr	Undecanoic acid
**5b**		L-(+)-Tat	Octyl amine
**6a**	L-Orn	L-Ser	Undecanoic acid
**6b**			Dodecanoic acid
**6c**		D-Ser	Undecanoic acid
**6d**			Dodecanoic acid
**7**		L-Thr	Undecanoic acid
**8**		L-(+)-Tat	Octyl amine

**Table 2:** The chemical properties and antifungal activity levels of the Bk analogues.

Entry	Compounds	Found ESI-MS	Calcd. ESI-MS	PSA ^a^	logP ^a^	*t*_R_ ^b^	MIC ^d,e^ (mg/mL)
1	**1**	1098.18	-------	422.9	−10.39	---	1.6
2	**2**	1019.12	-------	355.3	−8.57	---	25
3	**3a**	1092.23	1092.53	388.5	−10.65	9.3	>200
4	**3b**	1106.29	1106.55	387.6	−10.24	11.7	100
5	**3c**	1092.27	1092.53	389.1	−10.65	9.4	>200
6	**3d**	1106.29	1106.55	394.3	−10.24	11.8	>200
7	**4a**	1106.29	1106.55	381.1	−10.34	10.4	>200
8	**5a**	1080.23	1080.50	406.4	−12.28	5.3	>200
9	**3e**	1092.27	1092.53	380.8	−10.65	9.4	>200
10	**3f**	1106.29	1106.55	383.2	−10.24	11.8	200
11	**3g**	1092.27	1092.53	385.6	−10.65	9.6	>200
12	**3h**	1106.35	1106.55	395.7	−10.24	11.8	>200
13	**4b**	1106.35	1106.55	382.5	−10.34	10.7	>200
14	**5b**	1080.28	1080.50	381.6	−12.28	6.4	>200
15	**6a**	1106.43	1106.18	381.4	−10.21	9.7	>200
16	**6b**	1120.23	1120.56	395.0	−9.80	12.1	>200
17	**6c**	1106.39	1106.18	379.9	−10.21	--- ^c^	>200
18	**6d**	1120.32	1120.56	384.4	−9.80	11.2	>200
19	**7**	1120.25	1120.56	386.8	−9.90	10.6	>200
20	**8**	1094.29	1094.50	396.2	−11.84	5.8	>200

^a^ PSA and ClogP were calculated using SPARTAN’18 (Wavefunction); ^b^ 30–70% MeCN/H_2_O for 30 min; ^c^ **6c** had low solubility and the peak was not detected clearly in HPLC; ^d^ MIC values for Bk analogues are shown against *S. cerevisiae*, ^e^ MIC values of Bk analogues were determined as over MIC_90_.

**Table 3:** The chemical yields of each step for the corresponding peptides.

Entry	Compounds	A (%) ^a^	B (%) ^b^	C (%) ^c^	D (%) ^d^
1	**3a**	81	43	7	3
2	**3b**	quant.	32	12	4
3	**3c**	quant.	45	8	4
4	**3d**	quant.	27	23	6
5	**4a**	74	45	6	2
6	**5a**	quant.	26	29	8
7	**3e**	quant.	34	29	10
8	**3f**	quant.	42	31	13
9	**3g**	quant.	25	23	6
10	**3h**	96	45	37	16
11	**4b**	quant.	67	34	23
12	**5b**	quant.	74	33	25
13	**6a**	56	16	20	2
14	**6b**	63	33	9	2
15	**6c**	quant.	77	5	4
16	**6d**	86	55	11	5
17	**7**	41	82	10	3
18	**8**	39	49	16	3

^a^ The chemical yields from resin loading without further purification. ^b^ Cyclization efficiency from linear precursors after the purification by silica gel column chromatography. ^c^ Global deprotection of protected cyclic peptides after HPLC purification. ^d^ Overall yields from resin loading.

**Table 4:** The potentiation activity of **3g**.

	3g ^a^	G418	3g ^a^	zeocin
MIC ^b^ (mg/mL)	NI	25	NI	3.13
	12.5		3.13	

Note: ^a^ **3g** was used at a rate of 100 mg/mL; ^b^ these assays were attempted three times.

## Data Availability

Data sharing is not applicable to this article.

## Supplementary Materials

The following supporting information can be downloaded. Figure S1: HPLC profiles of synthetic peptides. HPLC conditions: detection at 220 nm.

## Sample Availability

Samples of the compounds are not available upon request from the authors.

## Abbreviations

SPPS: solid-phase peptide synthesis; DMF, dimethyl formamide; DIPEA, diisopropylethylamine; DIPC, diisopropylcarbodiimide; HOBt, 1-hydroxybenzotriazole; Fmoc, 9-fluorenylmethyloxycarbonyl; Alloc, allyloxycarbonyl; Boc, t-butyloxycarbonyl; TIPS, triisopropylsilane; BOP, benzotriazole-1-yloxy)tris(dimethylamino)phosphonium hexafluorophosphate; 2-CT, 2-chlorotrityl; HFIP, 1,1,1,3,3,3-hexafluoropropan-2-ol; TFA, trifluoroacetic acid; MIC, minimum inhibitory concentration.
