# Supplementary material for: The Hydrophobicity and Antifungal Potentiation of Burkholdine Analogues
Source: Molecules. 2022 Feb 10;27(4):1191. doi: 10.3390/molecules27041191 (PMC8877233; doi:10.3390/molecules27041191)
Supplement: Supplementary file 1 [file molecules-27-01191-s001.zip › molecules-1563902-supplementary.pdf]

Supporting information

The hydrophobicity and antifungal potentiation of burkholdine analogues

Hiroyuki Konno,[1,*] Mio Sasaki,[1] Hinata Sano,[1] Keima Osawa,[1] Kazuto Nosaka,[2] Shigekazu Yano[1]

[1]Department of Biological Engineering, Graduate School of Science and Engineering, Yamagata University, Yonezawa, Yamagata 992-8510, Japan.

[2]Faculty of Pharmaceutical Science, Mukogawa Women's University, Nishinomiya, Hyogo 663-8179, Japan.

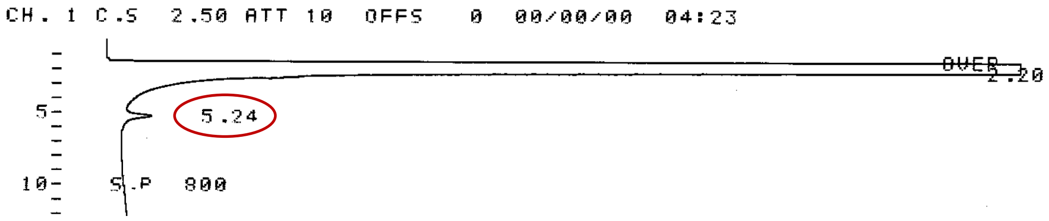

HPLC chart of **3a** [flow rate: 1 mL/min, 0-30 min, 30-60% MeCN/H_2O, t_R = 5.2]

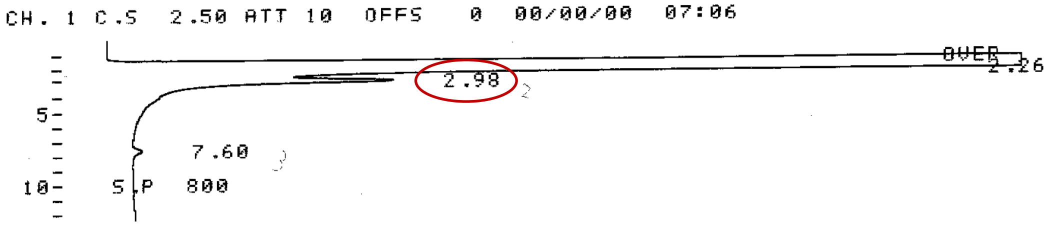

HPLC chart of **3b** [flow rate: 1 mL/min, 0-30 min, 40-70% MeCN/H_2O, t_R = 3.0]

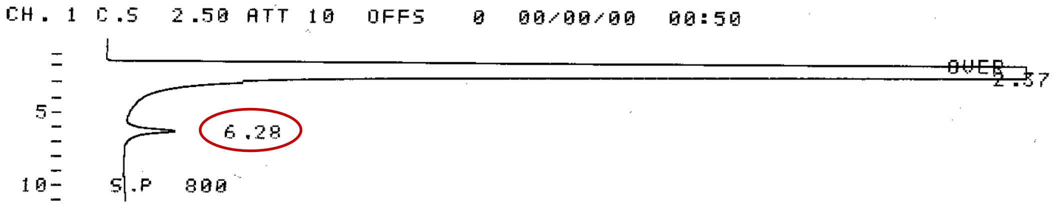

HPLC chart of **3c** [flow rate: 1 mL/min, 0-30 min, 30-60% MeCN/H_2O, t_R = 6.3]

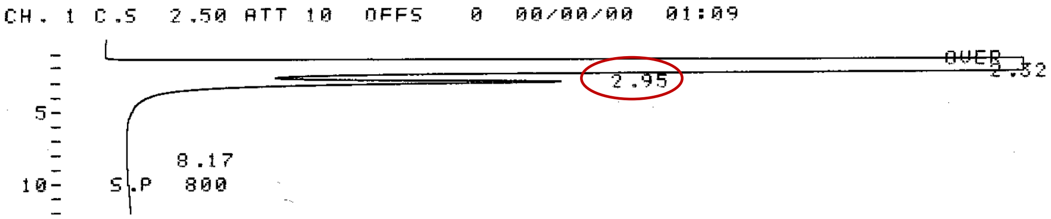

HPLC chart of **3d** [flow rate: 1 mL/min, 0-30 min, 40-70% MeCN/H_2O, t_R = 3.0]

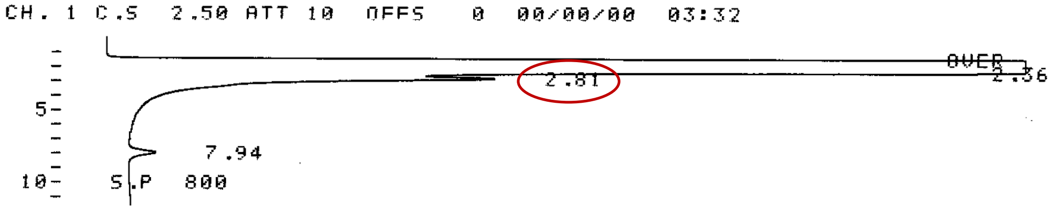

HPLC chart of **4a** [flow rate: 1 mL/min, 0-30 min, 40-70% MeCN/H_2O, t_R = 2.8]

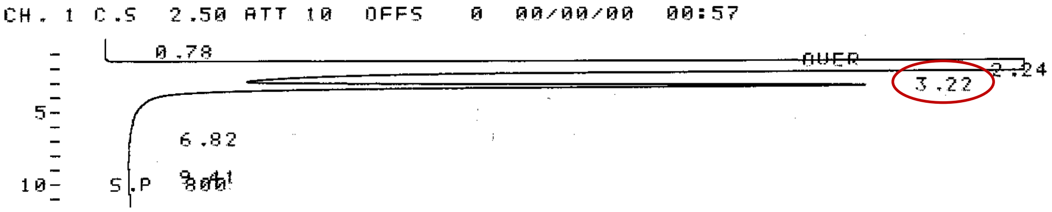

HPLC chart of **5a** [flow rate: 1 mL/min, 0-30 min, 30-60% MeCN/H_2O, t_R = 3.2]

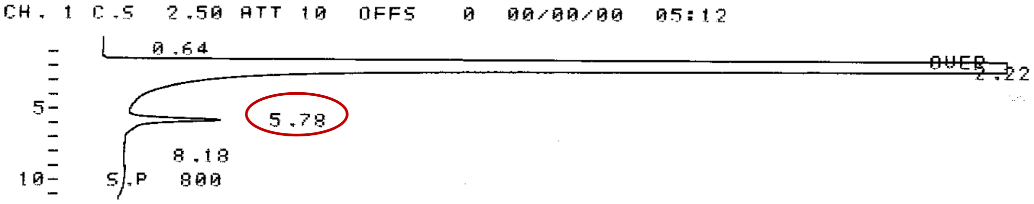

HPLC chart of **3e** [flow rate: 1 mL/min, 0-30 min, 30-60% MeCN/H_2O, t_R = 5.8]

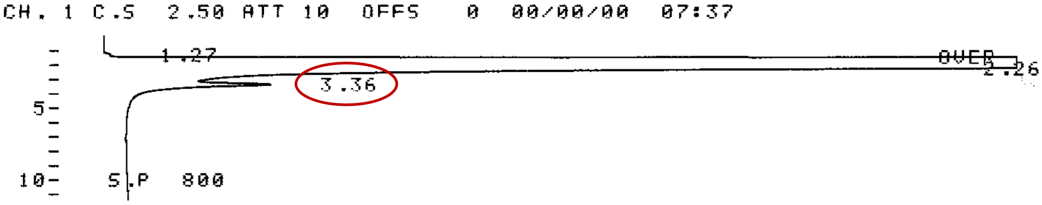

HPLC chart of **3f** [flow rate: 1 mL/min, 0-30 min, 40-70% MeCN/H_2O, t_R = 3.4]

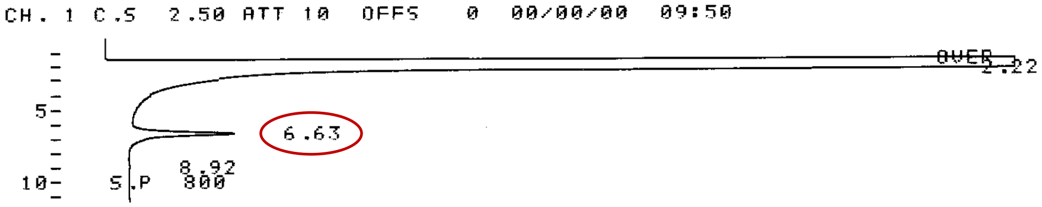

HPLC chart of **3g** [flow rate: 1 mL/min, 0-30 min, 30-60% MeCN/H_2O, t_R = 6.6]

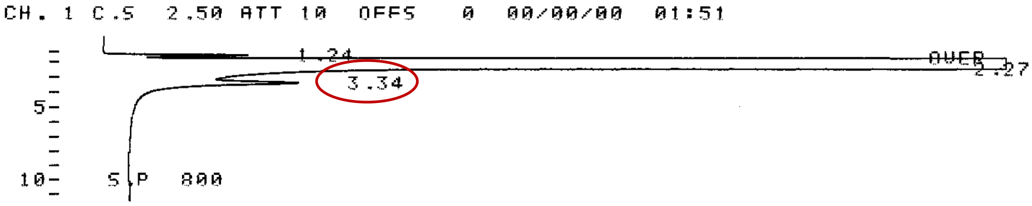

HPLC chart of **3h** [flow rate: 1 mL/min, 0-30 min, 40-70% MeCN/H_2O, t_R = 3.3]

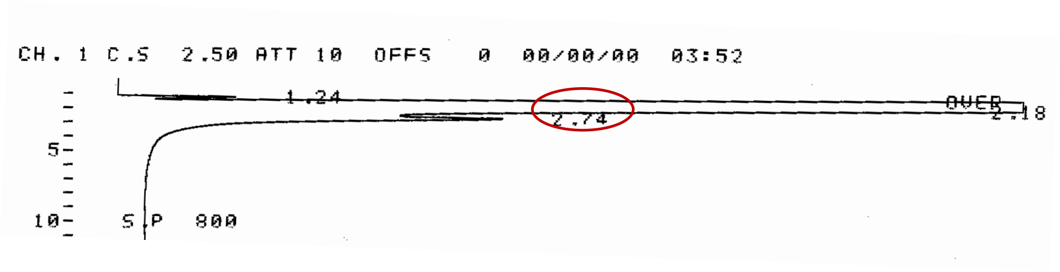

HPLC chart of **4b** [flow rate: 1 mL/min, 0-30 min, 30-60% MeCN/H_2O, t_R = 3.2]

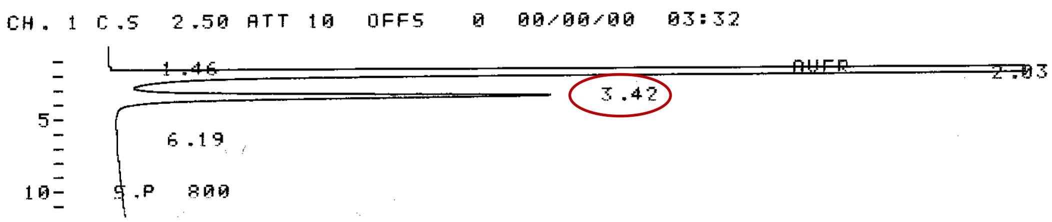

HPLC chart of **5b** [flow rate: 1 mL/min, 0-30 min, 30-60% MeCN/H_2O, t_R = 3.4]

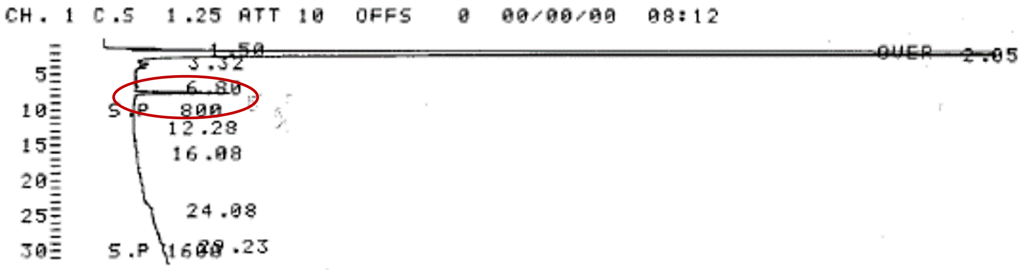

HPLC chart of **6a** [flow rate: 1 mL/min, 0-30 min, 30-60% MeCN/H_2O, t_R = 7.6]

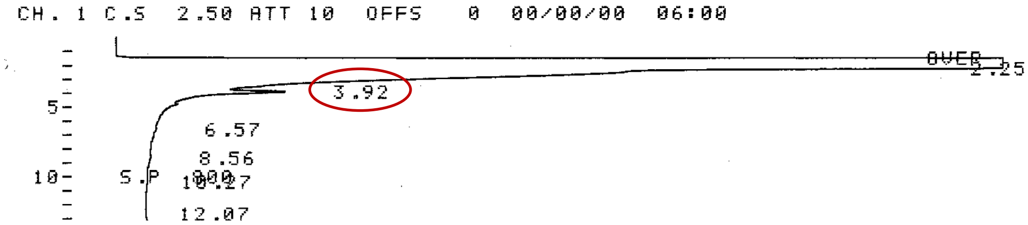

HPLC chart of **6b** [flow rate: 1 mL/min, 0-30 min, 40-70% MeCN/H_2O, t_R = 3.9]

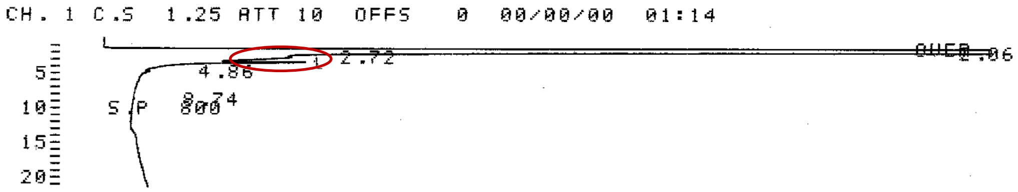

HPLC chart of **6d** [flow rate: 1 mL/min, 0-30 min, 40-70% MeCN/H_2O, t_R = 3.5]

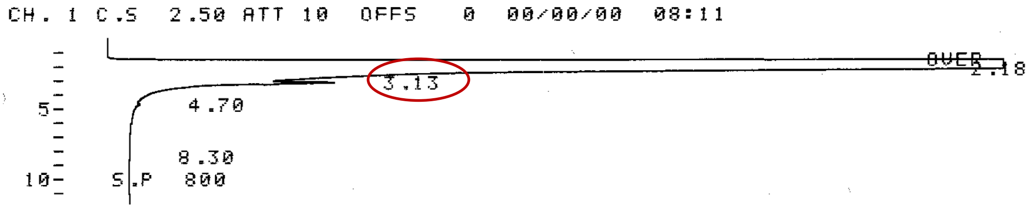

HPLC chart of **7** [flow rate: 1 mL/min, 0-30 min, 40-70% MeCN/H_2O, t_R = 3.1]

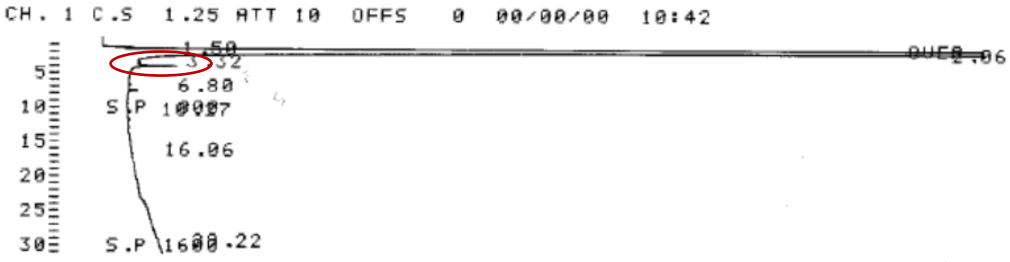

HPLC chart of **8** [flow rate: 1 mL/min, 0-30 min, 30-60% MeCN/H_2O, t_R = 3.7]

Figure. S1. HPLC profiles of synthetic peptides. HPLC conditions: detection at 220 nm.
